# Superficial Collagen Fibril Modulus and Pericellular Fixed Charge Density Modulate Chondrocyte Volumetric Behaviour in Early Osteoarthritis

**DOI:** 10.1155/2013/164146

**Published:** 2013-03-24

**Authors:** Petri Tanska, Siru M. Turunen, Sang Kuy Han, Petro Julkunen, Walter Herzog, Rami K. Korhonen

**Affiliations:** ^1^Department of Applied Physics, University of Eastern Finland, P.O. Box 1627, 70211 Kuopio, Finland; ^2^Fischell Department of Bioengineering, A.J. Clark School of Engineering, University of Maryland, College Park, MD 20742, USA; ^3^Department of Clinical Neurophysiology, Kuopio University Hospital, 70211 Kuopio, Finland; ^4^Human Performance Laboratory, Faculty of Kinesiology, University of Calgary, Calgary, AB, Canada T2N 1N4; ^5^Mechanical and Manufacturing Engineering, Schulich School of Engineering, University of Calgary, Calgary, AB, Canada T2N 1N4

## Abstract

The aim of this study was to investigate if the experimentally detected altered chondrocyte volumetric behavior in early osteoarthritis can be explained by changes in the extracellular and pericellular matrix properties of cartilage. Based on our own experimental tests and the literature, the structural and mechanical parameters for normal and osteoarthritic cartilage were implemented into a multiscale fibril-reinforced poroelastic swelling model. Model simulations were compared with experimentally observed cell volume changes in mechanically loaded cartilage, obtained from anterior cruciate ligament transected rabbit knees. We found that the cell volume increased by 7% in the osteoarthritic cartilage model following mechanical loading of the tissue. In contrast, the cell volume decreased by 4% in normal cartilage model. These findings were consistent with the experimental results. Increased local transversal tissue strain due to the reduced collagen fibril stiffness accompanied with the reduced fixed charge density of the pericellular matrix could increase the cell volume up to 12%. These findings suggest that the increase in the cell volume in mechanically loaded osteoarthritic cartilage is primarily explained by the reduction in the pericellular fixed charge density, while the superficial collagen fibril stiffness is suggested to contribute secondarily to the cell volume behavior.

## 1. Introduction

Osteoarthritis (OA) is a progressive joint disease, in which the tissue composition, structure, and mechanical properties are altered significantly [1–3]. Anterior cruciate ligament transection (ACLT) in rabbits is a common and well-defined model of early OA. Alterations in knee joint cartilage following ACLT have been shown to mimic changes in human OA cartilage both structurally and biologically, although the disease progression is faster in rabbits than in humans [4, 5]. During the early stages of OA, the superficial proteoglycan (PG) content and collagen fibril orientation are reported to be altered, while the collagen content remains relatively unchanged [1–3, 6] or is slightly reduced [7]. In addition, it has been suggested that the pericellular matrix (PCM) is also changed in early OA [8–10]. These changes in ECM and PCM properties and structure lead to altered osmotic and mechanical environments for the chondrocytes [11–15], possibly altering chondrocyte volume, morphology, and biosynthesis [6, 11–13, 15, 16]. The ability of the PCM to protect cells may also become weakened in OA [9, 10, 17–21], exposing cells to malfunction and death.

It has been reported that experimental chondrocyte deformations in response to mechanical load substantially differ in ACL transected joint compared to contralateral and normal joints. Especially, the cell volume has been shown to increase in mechanically loaded ACL transected joint cartilage, while it has been reported to decrease in contralateral and normal joint cartilage [22, 23]. It is speculated that the increase in cell volume results primarily from the altered PCM properties [8–10, 18, 20, 24–26]. However, the effect of changes in tissue properties on cell volumetric behaviour cannot be easily quantified using experimental techniques. Finite element (FE) analysis can be used to evaluate specific properties of articular cartilage on chondrocyte function [3, 9, 10, 18, 20, 24–27]. Specifically, fibril-reinforced biomechanical FE models can differentiate between the effects of changes in different cartilage constituents (i.e., collagen network, fixed charge density (FCD), and interstitial fluid) in the ECM and PCM on cell responses and cell-tissue interactions [10, 18, 20, 24–27]. 

The aim of this study was to find the cause for the altered cell deformation behaviour in healthy cartilage and cartilage in the early stages of OA using fibril-reinforced computational modeling. The models including ECM, PCM, and cell accounted for depth-dependent changes in collagen content, FCD, and collagen orientation, as obtained from Fourier Transform InfraRed (FTIR) microspectroscopy, digital densitometry (DD), and polarized light microscopy (PLM) of the experimentally tested samples. We simulated theoretically the experimentally used loading protocol and analyzed the corresponding changes in cell volume and morphology. Furthermore, we conducted parametric analysis to quantify the individual contributions of the FCD, collagen, and water of the PCM and ECM on the changes in cell volume. We hypothesized that primarily the properties of the collagen fibrils in the ECM and the amount of fixed charges in the PCM affect cell volumetric behaviour. As a result, this study provides better understanding of the interactions between chondrocytes, PCM, and ECM in early OA.

## 2. Materials and Methods

### 2.1. Experimental Analysis of Cell Volume

In previous experimental studies, patellae from skeletally mature New Zealand white rabbits were harvested 4 weeks [22] and 9 weeks [23] after ACLT. The samples were taken from the experimental and contralateral (CNTRL) joints [22, 23]. Surgical procedure was carried out according to the guidelines of the Canadian Council on Animal Care and was approved by the committee on Animal Ethics at the University of Calgary.

The cartilage samples were stained with Dextran (Invitrogen, Molecular Probes, OR, USA, final concentrations 0.8 mg/mL [23] and 4.8 mg/mL [22]) for four to eight hours at 4°C, washed in phosphate buffered saline (PBS) in order to remove excess Dextran, and fixed in a sample holder. Then, the samples were kept in PBS. For more details, see [22, 23].

A laser scanning microscope (Zeiss LSM 510 META, Carl Zeiss, Inc., Germany) with conventional confocal and dual-photon modes combined with an indentation system (indenter dia. 2 mm) was used for simultaneous cell imaging and mechanical indentation of the samples obtained 9 [23] and 4 [22] weeks after ACLT. Calibration of the system was done by imaging polystyrene microspheres (Polysciences Inc., Warrington, PA, USA, diameter 5.93 ± 0.05) in Dextran-stained agarose gel. A 2 MPa pressure was first applied on the middle of the sample at a speed of ~6 *μ*m/s [23] and ~10 *μ*m/s [22]. This was followed by force relaxation; that is, the displacement was held constant for 20 minutes. For the analysis of cell volume and morphology, image stacks were captured at 0.5 *μ*m increments up to 60 *μ*m in depth from the cartilage surface, before and after the mechanical loading. Image thresholds were defined for each cell individually using the median value of the intensity histogram of the image [22, 23] and 3D images of the cells were reconstructed. Local axial and transversal ECM strains were calculated according to the previous studies (*ε* = (*d* − *d*_0_)/*d*_0_, where *d*_0_ and  *d*  are the distances between cells before and after loading, respectively) [22, 23, 28]. System calibration, 3D image reconstruction, cell volume and morphology analysis, and determination of local ECM srains are presented in more detail in previous studies [22, 23, 29].

### 2.2. Microscopic Analysis of Tissue Structure and Composition

Following indentation testing, composition (collagen and PG content), and collagen orientation of the cartilage samples were determined using FTIR microspectroscopy, DD, and PLM, respectively [22]. Patellae were fixed in formalin, decalcified in EDTA, dehydrated, and treated with xylene before embedding in paraffin (for details, see [30–32]). Microscopic sections (three sections per sample, thickness 5 *μ*m for FTIR, and 3 *μ*m for DD and PLM [22]), obtained from the same area where the mechanical loading of the cartilage occurred, were cut perpendicularly to the cartilage surface. The sections for PLM and FTIR were deparaffinized and PGs were removed with hyaluronidase digestion (1000 U/mL hyaluronidase, Sigma-Aldrich, St. Louis, MO, USA). For DD the samples were stained with safranin-O. Sections for FTIR were placed on ZnSe windows, while those for PLM and DD were placed on standard microscopic slides. FTIR, DD, and PLM techniques for the analysis of cartilage structure and composition have been presented in detail in several other studies [3, 7, 30, 31, 33–38]; thus, only a brief description of these methods is given here.

Spatial PG content of the samples was determined with DD (Photometrics CH250 ltd., Tucson, Arizona, USA) by quantifying the optical density (OD) of safranin-O stained sections [22, 33–37]. Absorbance images (4x magnification, 700 ms exposure time) were captured from the cartilage surface to the cartilage-bone interface from a manually drawn ~400 *μ*m wide rectangular profile. Profiles were averaged horizontally to indicate the depth-dependent PG content of the samples.

### 2.3. Finite Element Analysis

An axisymmetric finite element (FE) model was constructed with fibril-reinforced poroelastic swelling properties [24, 39]. Briefly, in the fibril-reinforced model, articular cartilage is assumed to be a biphasic material consisting of a nonfibrillar (PGs and fluid) and a fibrillar (collagen network) matrix. The fibrillar matrix is composed of organized primary and randomly organized secondary fibrils. The primary fibrils possess an arcade-like orientation; in the superficial zone fibrils are parallel to the surface; in the middle zone they bend toward the deep zone where the fibrils are perpendicular to the cartilage surface [40]. At each integration point, the initial fibril orientation is expressed by a unit vector$\;\;\left({\vec{e}}_{f,0}\right)$. Following tissue deformation, the fibril network is realigned and new fibril orientations $\left({\vec{e}}_{f}\right)$ are calculated using the following equation:

(1) $$\begin{array}{c} {\vec{e}}_{f}=\;\frac{F\cdot {\vec{e}}_{f,0}}{||F\cdot {\vec{e}}_{f,0}||}, \end{array}$$

where **F** is the deformation gradient tensor. The collagen network was modeled using elastic fibrils resisting only tension; thus, the fibril stress (*σ*_*f*_) is given by

(2) $$\begin{array}{c} \sigma_{f}=\{\begin{array}{cc} E_{f}\varepsilon_{f}, & \varepsilon_{f}\geq 0,\\ 0, & \varepsilon_{f}<0, \end{array} \end{array}$$

where *E*_*f*_ is the fibril network modulus and *ε*_*f*_ is the fibril strain. The primary and secondary fibril stresses can be expressed as

(3) σf,pri  =ρc,totCσf (for primary fibrils),σf,sec  =ρc,totσf (for secondary fibrils),

where  *σ*_*f*,pri_ and *σ*_*f*,sec_  are the stresses of primary and secondary fibrils, respectively; *C* is a positive constant describing the density ratio between primary and secondary fibrils; and *ρ*_*c*,tot_ is the depth-dependent total collagen fraction per total solid volume [39]. The number of fibrils in each integration point was set to *n*_pri_ = 2 and *n*_sec_ = 7 [39].

The nonfibrillar matrix was implemented as a nonlinear neo-Hookean porohyperelastic material. The neo-Hookean stress (**σ**_*m*_) can be expressed as

(4) $$\begin{array}{c} \sigma_{m}=K\frac{\ln\left(J\right)}{J}I+\frac{G}{J}\left(F\cdot F^{T}-J^{2/3}I\right), \end{array}$$

where *K* and *G* are bulk and shear moduli, *J* = det⁡(**F**), and  **I**  is unity tensor. Permeability *k* was implemented as in previous studies [18, 39, 41] and assumed to be void-ratio dependent according to [42]

(5) $$\begin{array}{c} k=\;k_{0}{\left(\frac{1+e}{1+e_{0}}\right)}^{M}, \end{array}$$

where *k*_0_ is the initial permeability; *e* and *e*_0_ are the current and initial void ratios, respectively; and *M* is a positive constant. 

The fibril-reinforced model included also osmotic swelling and chemical expansion stresses to account for tissue swelling. The Donnan osmotic swelling pressure gradient at equilibrium can be determined as, [43],

(6) $$\begin{array}{c} \Delta \pi =\phi_{\mathrm{int}}RT\left(\sqrt{c_{F}^{2}+\;4\frac{{\left(\gamma_{\hat{\text{e}}\text{xt}}^{\pm }\right)}^{2}}{{\left(\gamma_{\mathrm{int}}^{\pm }\right)}^{2}}}\right)-2\phi_{\text{ext}}RTc_{\text{ext}}, \end{array}$$

where *ϕ*_int⁡_, *ϕ*_ext_, *γ*_int⁡_^±^, and $\gamma_{\hat{\text{e}}\text{xt}}^{\pm }\;\;$are internal and external osmotic coefficients and internal and external activity coefficients, respectively; *c*_ext_ is external salt concentration (0.15 M); *R* is the molar gas constant (8.3145 J/mol K); *T* is the absolute temperature (293 K). The chemical expansion stress can be expressed as, [44],

(7) $$\begin{array}{c} T_{c}=a_{0}c_{F}\exp\left(\kappa \frac{\gamma_{\hat{\text{e}}\text{xt}}^{\pm }}{\gamma_{\mathrm{int}}^{\pm }}\sqrt{c^{-}\left(c^{-}+c_{F}\right)}\right), \end{array}$$

where *a*_0_ and *κ* are material constants [39] and *c* ^−^ is the mobile anion concentration. 

Total stress (**σ**_*t*_)  in the fibril-reinforced poroelastic swelling model is given as follows, [39]:

(8) $$\begin{array}{c} \sigma_{t\;}=\sum_{i=1}^{\text{tot}f}\sigma_{f}^{i}+\sigma_{m}-T_{c}I-\;\Delta \pi I-\;\mu_{f}I, \end{array}$$

where  **σ**_*f*_^*i*^ is the individual fibril stress, tot *f* is the total number of fibrils, and *μ*_*f*_ is the electrochemical potential of water [44].

FTIR was used to determine the spatial collagen content of cartilage by observing the characteristic infrared absorption spectrum of cartilage [3, 7, 22, 23, 38, 45, 46]. Samples were imaged (spectral resolution = 4 cm^−1^, pixel size = 6.25 *μ*m) from the cartilage surface to the subchondral bone (500 *μ*m wide rectangular area). Obtained spectra were offset-corrected by subtracting the minimum value of the spectra, and the average depth-wise collagen content was calculated by averaging the integrated absorbance values of amide I peak (1585–1720 cm^−1^) [3, 7] in a plane parallel to the cartilage surface. All FTIR measurements were performed using a PerkinElmer Spectrum Spotlight 300 FTIR-imaging system (Perkin Elmer, Waltham, MA, USA) in transmission mode.

PLM was used to analyze depth-dependent collagen fibril orientation [22, 30, 31, 33]. Measurements were conducted on unstained sections (width ~400 *μ*m, height from the surface to the cartilage-bone interface) by using a Leitz Ortholux II POL polarized light microscope (Leitz, Wetzlar, Germany) and a Peltier-cooled, high-performance CCD camera (Photometrics SenSys, Roper Scientific, Tucson, AZ, USA). Images were recorded with a 4x magnification and an exposure time of 800 ms. Finally, a depth-wise collagen fibril orientation map was calculated with Stokes parameters [31].

#### 2.3.1. ACL Transected and Contralateral Models

**Figure 1:**
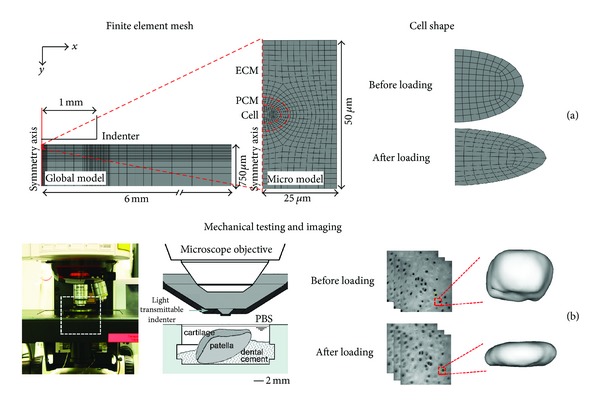
Finite element model (a) and corresponding microscopy and mechanical testing setup (b). Fixed patellar cartilage samples immersed in phosphate buffered saline (PBS) were imaged during mechanical loading. Image stacks were formed and cell volumes and dimensions calculated before and after loading (at steady state). Then, an axisymmetric global model and a micromodel were constructed. Cell volume and morphology before loading and at equilibrium were analyzed and compared with experimental results.

For the FE simulation of experimental measurements, a global model was constructed for an indentation geometry containing 1608 4-node axisymmetric porous elements (type CAX4P) and included only the ECM (Figure 1). The indenter was assumed rigid in the model. The movement of the symmetry axis was restricted in the lateral direction, while the bottom of the sample was fixed in the axial direction. At the edge of the cartilage and free cartilage surface (not in contact with the indenter), free fluid flow was allowed. At the initial position the indenter was not in contact with cartilage surface. After a free swelling step (tissue swelling due to (6) and (7)) the indenter was moved to contact with the cartilage surface. A frictionless contact was assumed between the cartilage surface and the indenter. The contact was followed by a 2 MPa compression step and a 20 min force relaxation period, as done in the experiments [22, 23]. The submodel, located in the superficial zone and driven by a nodal output (displacements and pore pressures) obtained from the global model, was constructed with a small portion of the ECM (398 elements). It included the PCM (80 elements) and cell (102 elements) (Figure 1). Cell height and width were determined directly from the experimental 3D reconstructions. The fibril-reinforced poroelastic swelling material was implemented for the ECM and PCM with user-defined material script (UMAT), while the fibrils were ignored in the cell. Simulations were performed with Abaqus 6.11-2 (Dassault Systémes, Providence, RI, USA).

**Figure 2:**
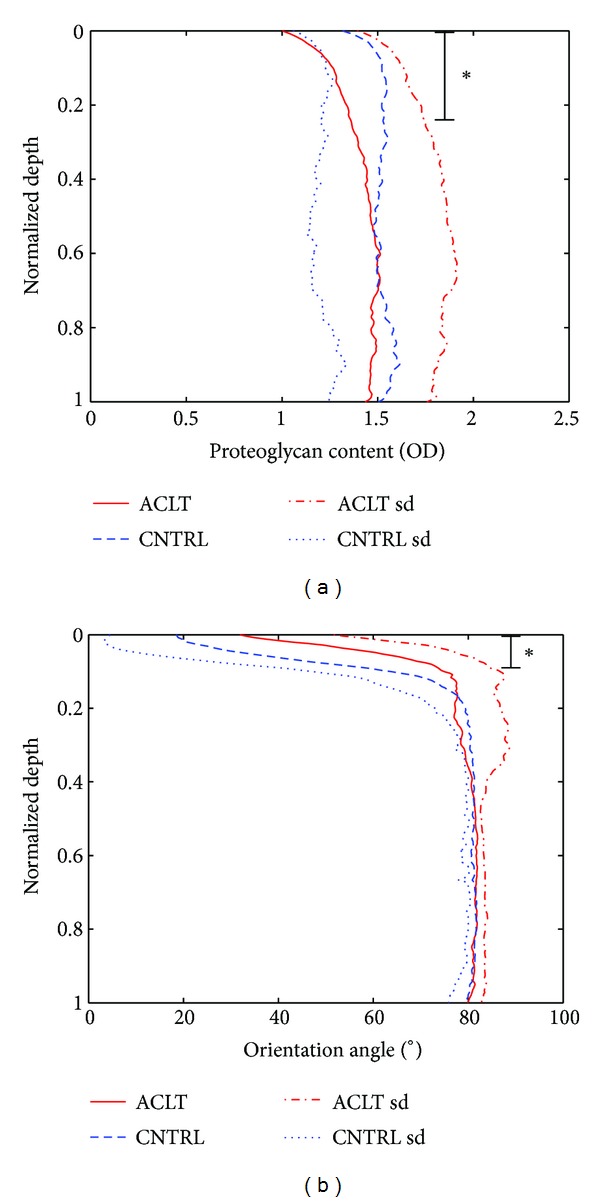
Microscopic analyses of the anterior cruciate ligament transected (ACLT) and contralateral (CNTRL) cartilage sections as a function of the normalized tissue depth (0 = surface, 1 = cartilage-bone interface) [22]. (a) Depth-dependent optical density (OD) representing proteoglycan content of the ACLT and CNTRL cartilage. (b) Depth-dependent collagen fibril orientation angle of the ACLT and CNTRL cartilage. Data is presented as mean ± S.D. *P < 0.05 between the groups.

First, the composition, structure, and mechanical parameters of contralateral joint cartilage were obtained from the microscopic and spectroscopic analyses and the literature, and implemented into the model (Figure 2, Tables 1 and 2, CNTRL model). The zonal thicknesses, obtained from the PLM data of the contralateral joint cartilage (Figure 2(b)), were 7%, 18%, and 75% of cartilage thickness for the superficial, middle, and deep zones, respectively [47]. The water fraction, collagen fraction, and FCD values were obtained from earlier studies [24, 48]. The PCM collagen stiffness was assumed to be 10% of that of the ECM and the fluid fraction was assumed constant, similarly as in earlier studies [10, 18]. The FCD of the PCM was assumed to be higher than that of the ECM (1.27 times the FCD of the ECM) [10, 18] and the collagen network direction in the PCM was parallel to the cell membrane [21]. The FCD of the cell was assumed to be lower than that of the ECM [49] and it was similar as used in earlier studies [10, 18, 50]. Permeability was assumed to be nonlinear, void-ratio dependent (*M* = 8.1, (5)) in the ECM [10, 18] and constant (*M* = 0, (5)) in the PCM and cell. A summary of the mechanical, structural, and compositional parameters of the ECM, PCM, and cell for the reference CNTRL model is presented in Table 1.

The changes in cartilage properties in early OA (i.e., reduced collagen fibril stiffness due to increased superficial fibrillation in the ECM and decreased superficial FCD), estimated from our 4-week experimental test group [22], were implemented into the ACLT model (Figure 2, Table 2, ACLT model). In addition, fibrillation of the PCM collagen fibrils by the reduced collagen fibril stiffness, decreased FCD content in the PCM and increased water content in the PCM, and ECM, presumably present in the ACLT (early OA) cartilage [1–4], were also implemented into the ACLT model. However, consistent with our experimental measurements [22], collagen content (see (3)) was not changed in the ACLT model. The CNTRL and ACLT models were compressed as in the experiments explained above. Then, the normalized cell volume was analyzed from both models and compared with the experimental results. In addition to the cell volume behavior, local axial and transversal ECM strains, and cell morphology obtained from the experimental studies [22, 23], were compared with the results from the FE simulations.

#### 2.3.2. Parametric Studies

*Experimental Loading Protocol*. In order to clarify the importance of the ECM and PCM properties on the cell volume for experimentally applied loading protocol, sensitivity analyses were conducted. The effects of the alterations in the ECM collagen fibril stiffness, FCD, and fluid fraction on the cell volume were explored, while the PCM properties were kept unchanged (Table 2, parametric ECM model). Since the PCM properties were hypothesized to further modulate the cell volume, but they could not be obtained directly from the experiments, a sensitivity analysis of the importance of PCM properties (i.e., varying PCM collagen fibril stiffness, FCD, and fluid fraction) on the cell volume was further conducted (Table 2, parametric CNTRL PCM and ACLT PCM models). The effect of the PCM properties in the CNTRL and ACLT models was simulated with and without the chemical expansion stress (see (7)). Finally, the effect of the pericellular FCD after free swelling step on chondron properties (cell volume, average fibril strain in the PCM, and average osmotic pressure in the PCM) was investigated.

*Constant Strain.* Since a major decrease in the collagen fibril modulus of the ECM increased tissue strain substantially by using the experimental loading protocol, the effect of the ECM and PCM properties on the normalized cell volume was also parametrically analyzed for constant tissue strain (*ε* = 16%) as produced in the experiments [23]. This was done by varying one of the ECM or PCM matrix components individually (collagen stiffness, FCD, fluid fraction) while keeping the other parameters constant (Table 2, Parametric ECM model and Parametric PCM models 1–4).

### 2.4. Statistical Analysis

All experimental data is presented as mean ± standard error of mean. A one-way ANOVA was applied in the parameter comparison between the groups (ACLT, contralateral). The structural and compositional differences between the samples were evaluated with Wilcoxon signed rank test. SPSS 17.0 (SPSS Inc., Chicago, IL, USA) was used for statistical analyses.

## 3. Results 

### 3.1. Experimental Results versus FE Simulations

**Figure 3:**
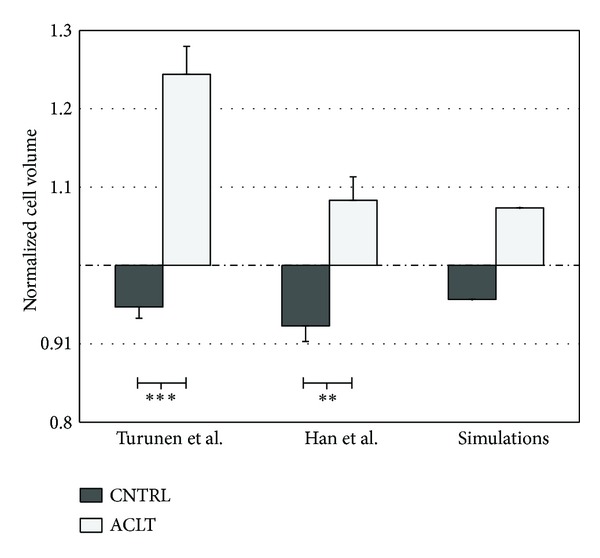
Experimentally detected normalized cell volume (cell volume after loading/cell volume before loading) in cartilage samples obtained from experimental (ACLT) and contralateral (CNTRL) joints at four weeks (Turunen et al. 2012) [22] and nine weeks (Han et al. 2010) [23] after anterior cruciate ligament transection. Results from the fibril-reinforced poroelastic models, representing contralateral (Table 2, CNTRL model) and ACLT cartilages (Table 2, ACLT model) are also shown (simulations). In all cases, a contact pressure of 2 MPa was applied, followed by a 1200 s force relaxation. Cell volume behaviour in the nonoperated normal group was similar to contralateral cartilage [23]. Experimental data is presented as mean ± standard error of mean. **P < 0.005, ***P < 0.001 between the groups.

The average superficial zone cell volume at steady state following tissue compression (2 MPa compression followed by 20 min force relaxation) was reduced by 5 ± 1% in contralateral and increased by 24 ± 4% in ACLT joint cartilage obtained 4 weeks after ACL transection [22]. In the cartilage samples obtained 9 weeks after ACL transection, the mechanical compression caused a 8 ± 2% decrease in the superficial tissue cell volume in contralateral and an 8 ± 3% increase in the superficial tissue cell volume in ACLT joint cartilage [23]. Volume changes between the contralateral and ACLT groups were significantly different in both 4 week (*P* < 0.001) and 9 week (*P* < 0.005) studies. In the FE model representing contralateral cartilage (Table 2, CNTRL model), a 4% decrease in superficial tissue cell volume was observed as a result of tissue compression. In the FE model representing ACLT joint cartilage (Table 2, ACLT model), cell volume increased by 7% (Figure 3).

As a result of tissue compression, change in cell height (axial cell strain) was 7 percentage points lower (*P* < 0.005) and change in cell width (transversal cell strain) was 9 percentage points higher (*P* < 0.001, Table 3, Turunen et al. [22]) in the ACLT joint cartilage (4 weeks after ACLT), compared to the contralateral group. In the ACLT joint cartilage obtained 9 weeks after ACLT, change in cell height was 6 percentage points (*P* < 0.005) lower and change in cell width was 5 percentage points higher (*P* < 0.001, Table 3, Han et al. [23]), compared to the contralateral group. In the FE model representing ACLT joint cartilage, tissue compression increased the change in cell height and width by 9 and 16 percentage points, respectively (Table 3, simulations), compared to the contralateral model.

In the ACLT joint cartilage obtained 4 weeks after ACLT, average local ECM strains as a result of tissue compression were 4 and 3 percentage points higher in axial and transversal directions, respectively, compared to the contralateral group (Table 3, Turunen et al. [22]). In the ACLT joint cartilage obtained 9 weeks after ACLT, average local ECM strains were 11 (*P* < 0.005) and 6 percentage points (*P* < 0.05) higher in axial and transversal directions, respectively, compared to the contralateral group (Table 3, Han et al. [23]). In the FE model representing the ACLT joint cartilage, local ECM strains were 9 and 5 percentage points higher in axial and transversal directions, respectively, compared to the contralateral group (Table 3, simulations).

### 3.2. Parametric Studies

#### 3.2.1. Experimental Loading Protocol

**Figure 4:**
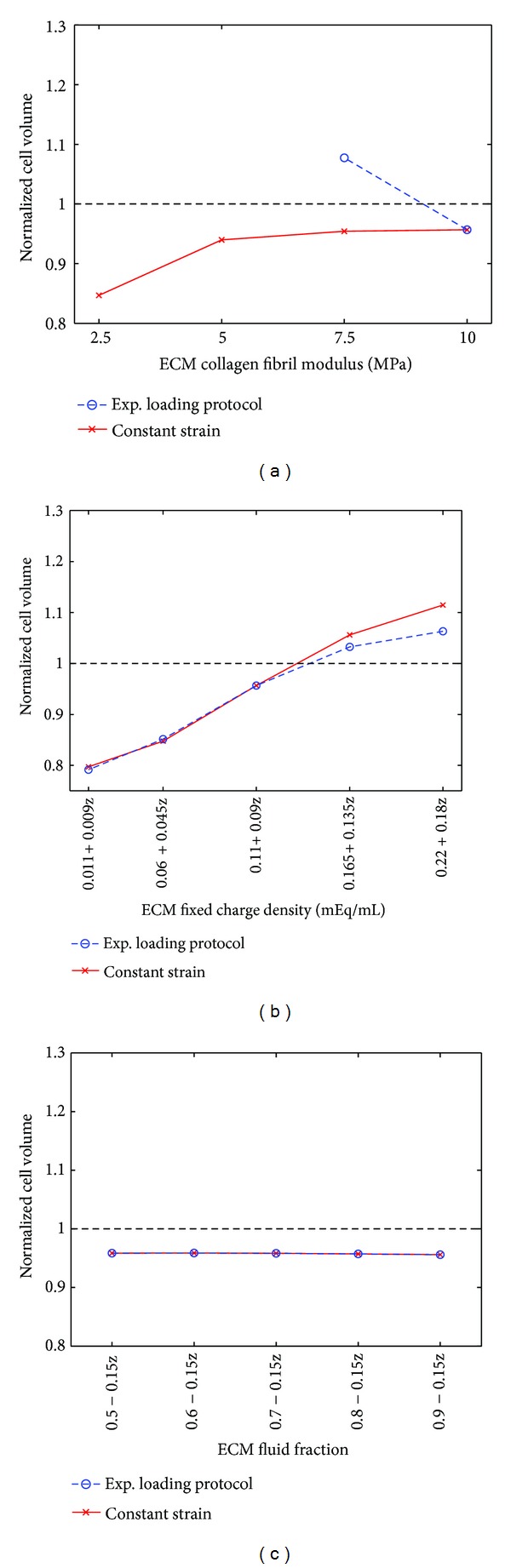
Normalized cell volume (cell volume after loading/cell volume before loading) in the simulations of the experimental loading protocol (blue, 2 MPa stress followed by force relaxation [22, 23]) and for the constant strain protocol (red, *ε* = 16%). The cell volume was analyzed as a function of the ECM (a) collagen fibril modulus, (b) fixed charge density, and (c) fluid fraction. Other model parameters were as in the CNTRL model (see Table 2, parametric ECM model). Above the horizontal line (- -) the cell volume is increased and under the line the cell volume is decreased.

Under the experimental loading protocol, the analysis of the effect of the ECM properties (Table 2, experimental loading protocol, parametric ECM model) showed that a decrease of the ECM collagen fibril stiffness from 10 MPa to 7.5 MPa caused a substantial increase in the normalized cell volume (Figure 4(a)). High amounts of the FCD in the ECM increased the cell volume, while a change in the fluid fraction of the ECM had a negligible effect on the normalized cell volume (Figures 4(b) and 4(c)).

**Figure 5:**
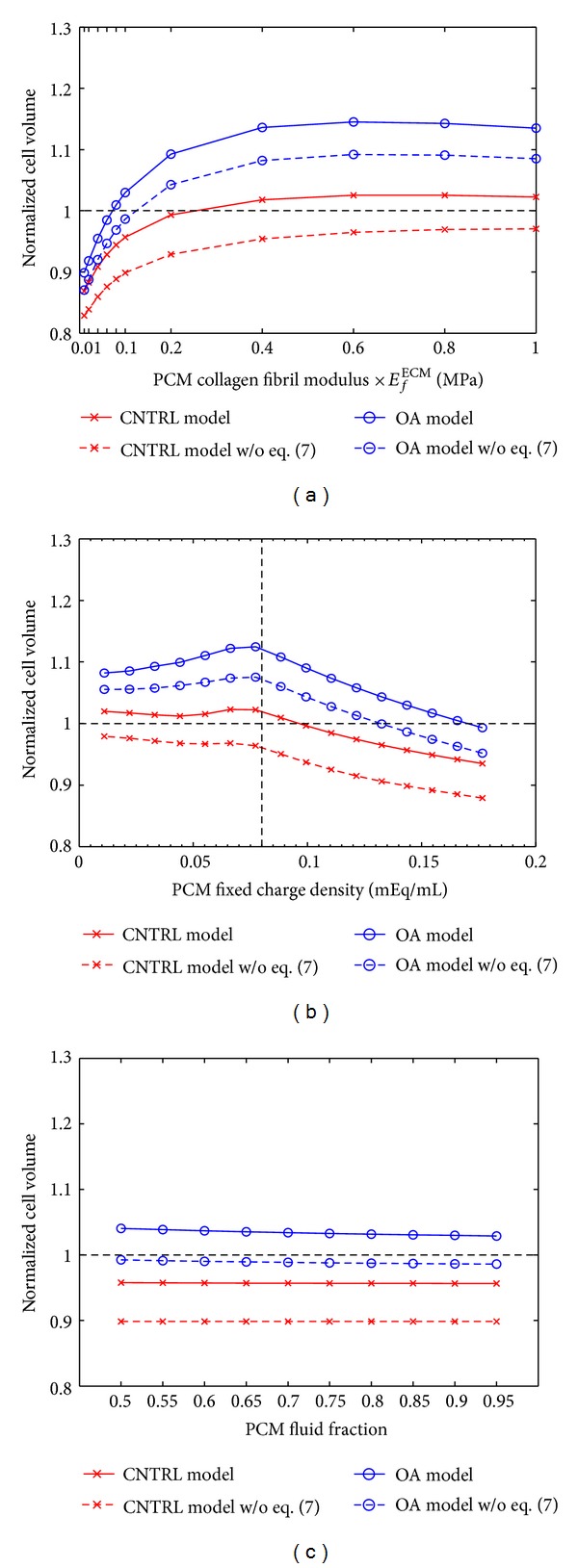
Normalized cell volume (cell volume after loading/cell volume before loading) in contralateral (CNTRL) and anterior cruciate ligament transected (ACLT) models as a function of the PCM (a) collagen fibril modulus, (b) fixed charge density, and (c) fluid fraction. Both CNTRL and ACLT models were compressed until a 2 MPa contact pressure was reached. This was followed by a 1200 s force relaxation. Model parameters are presented in Table 2 (CNTRL: Parametric CNTRL PCM model, ACLT: Parametric ACLT PCM model). Above the horizontal line (- -) the cell volume is increased and under the line the cell volume is decreased. Vertical line (- -) in (b) represents the fixed charge density of the cell in the superficial zone.

Additional analysis of the effect of the PCM properties in the ACLT model (Table 2, parametric ACLT PCM model) showed that an increase in the PCM collagen fibril stiffness with respect to the ECM fibril stiffness caused a further increase in the cell volume in the mechanically loaded tissue (Figure 5(a)). On the other hand, the maximum increase in the cell volume (12%) was observed by decreasing the pericellular FCD (Figure 5(b)). Changes in interstitial fluid fraction of the PCM had only a minor effect on the cell volume (Figure 5(c)). The same trends in the behavior of the normalized cell volume as a function of aforementioned compositional/structural changes were observed in the contralateral model (Figure 5 and Table 2, parametric CNTRL PCM model). In addition, those trends remained the same in the simulations without the chemical expansion stress (see (7), Figure 5), even though the cell volume increase was reduced in the ACLT model.

**Figure 6:**
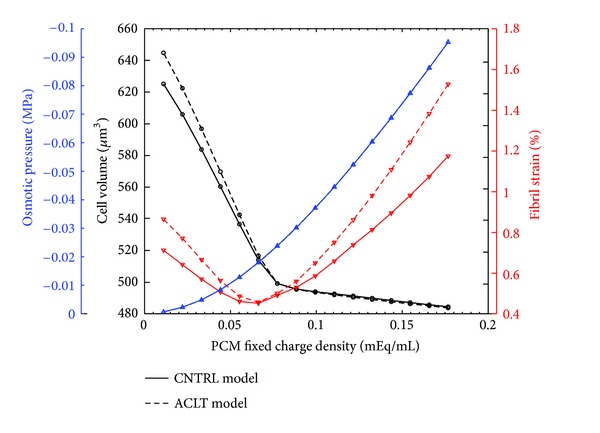
Cell volume (black), average pericellular fibril strain (red), and average pericellular osmotic pressure (blue) after the free swelling step in the contralateral (CNTRL) and anterior cruciate ligament transected (ACLT) models as a function of the pericellular fixed charge density. Osmotic pressure behavior is the same in both models. Note the change in the fibril strain and cell volume when the fixed charge density of the PCM is nearly equal to that of the chondrocyte (*c*_*F*_ = 0.08 mEq/mL).

After the free swelling step, a decrease in the pericellular FCD decreased the average osmotic pressure in the PCM and increased the absolute cell volume (Figure 6 and Table 2, parametric CNTRL PCM and parametric ACLT PCM models). The absolute cell volume was increased more in the ACLT model than in the CNTRL model when the pericellular FCD was lower than that of the cell (<0.08 mEq/mL), while the osmotic pressure remained the same in both models. When the pericellular FCD was reduced or increased from the value of ~0.07 mEq/mL, the average fibril strain in the PCM increased. The fibril strain was higher in the ACLT model compared to the CNTRL model.

#### 3.2.2. Constant Strain

In the model under a constant strain (16%; Table 2, constant strain, parametric ECM model) for 20 minutes, a decrease in the extracellular collagen fibril modulus and FCD decreased the normalized cell volume (Figures 4(a) and 4(b), respectively). Only high amounts of the FCD in the ECM caused an increased (>1) cell volume. A change in the ECM fluid fraction had a negligible effect on the normalized cell volume (Figure 4(c)).

**Figure 7:**
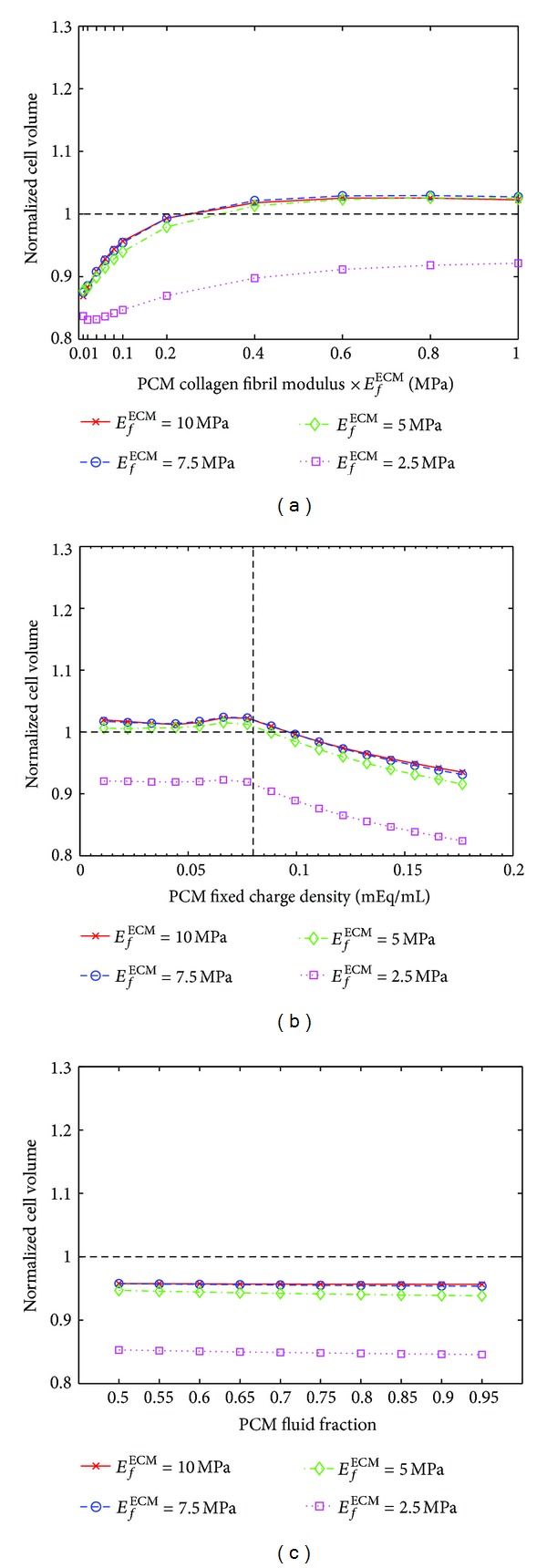
Normalized cell volume (cell volume after loading/cell volume before loading) as a function of the PCM (a) collagen fibril modulus, (b) fixed charge density, and (c) fluid fraction in the models with four different ECM collagen fibril modulus (*E*_*f*_^ECM^) values (Table 2, parametric PCM models 1–4). Other model parameters are as in the reference model. In all models, the strain was kept constant (*ε* = 16%). Above the horizontal line (- -) the cell volume is increased and under the line the cell volume is decreased. Vertical line (- -) in (b) represents the fixed charge density of the cell in the superficial zone.

The models under a constant strain predicted a decrease in the normalized cell volume when the PCM collagen fibril modulus was reduced (Figure 7(a)). In the models with *E*_*f*_^ECM^ = 5–10 MPa, the mechanical tissue compression increased the cell volume (>1) when the PCM collagen fibril modulus was greater than ~0.2 × *E*_*f*_^ECM^. This was not observed when *E*_*f*_^ECM^ was 2.5 MPa. In contrast, the normalized cell volume increased when the pericellular FCD was reduced (Figure 7(b)). In the models with *E*_*f*_^ECM^ = 5–10 MPa, the mechanical tissue compression caused an increase in the cell volume (>1) when the pericellular FCD was less than ~0.1 mEq/mL. This was not observed when *E*_*f*_^ECM^ was 2.5 MPa. Again, changes in the pericellular fluid fraction had only a minor effect on the normalized cell volume and it could not explain the increased cell volume (>1) as a result of tissue compression (Figure 7(c)).

## 4. Discussion

We investigated the cell volume behaviour in early OA cartilage using a fibril-reinforced, poroelastic swelling model. Compositional and structural parameters of the ECM were obtained from microscopic and spectroscopic analyses and literature, and the effects of changes in the ECM and PCM properties on cell volume and morphology were investigated. The FE models simulating normal and OA (reduced fibril network stiffness due to increased collagen fibril orientation angle [51], reduced FCD, and increased fluid fraction) cartilage reproduced experimentally detected cell volume changes in mechanically loaded cartilage. Increased local tissue strains as a result of reduced ECM collagen fibril stiffness and decreased pericellular FCD were shown to have the most significant effect on cell volume. 

The fibril-reinforced swelling model of OA cartilage was able to reproduce the experimentally observed increase in cell volume following mechanical tissue loading. The results specifically showed that increased superficial collagen network fibrillation in early OA, simulated by reduced tensile strength of the superficial zone collagens, increased global and local ECM strains; especially local transversal ECM strain was increased by 3, 6, and 5 percentage points in the 4- and 9-week experimental ACLT groups and ACLT FE model, respectively, compared to the contralateral groups. These increases of local transversal ECM strain can explain increases in cell width and volume following mechanical loading; 9-, 5-, and 16-percentage-point increase in width and 29-, 16-, and 11-percentage-point increase in volume were observed in the 4- and 9-week experimental ACLT groups and ACLT FE model, respectively, compared to the contralateral groups. Furthermore, a decrease in the pericellular FCD, presumably present in early OA, reduced the PCM swelling pressure and equilibrium stiffness [52, 53]. This further amplified the increase in cell volume isotropically, and up to 12% larger cell volume was simulated compared to the cell in the noncompressed cartilage. 

In contrast to our other hypothesis, decreases in the pericellular collagen network modulus could not explain the experimentally observed increases in cell volume following mechanical loading. When the collagen fibril modulus of the PCM was decreased, the stiffer fibrils in the ECM caused the PCM to experience larger tensile strain in the horizontal direction, thereby reducing cell elongation and volume. On the other hand, the pericellular collagen fibril stiffness may have an indirect effect on the cell volume increase. The collagen fibrils resist tissue expansion caused by the FCD, providing a meshwork for cartilage and holding it together and in shape [52–54]. If the pericellular FCD is reduced, tensile forces of this collagen meshwork are reduced, leading to decreased PCM stiffness. This may allow the cell to expand in mechanically loaded cartilage. It is possible that under dynamic/impact loading the effect of the pericellular collagen on the cell volume would be more apparent, because collagen fibrils are mainly responsible for the dynamic response of the articular cartilage.

During the free swelling step, the pericellular FCD modulated substantially the absolute cell volume, pericellular fibril strain, and osmotic pressure (Figure 6). As expected, the reduction in the pericellular FCD decreased the osmotic pressure in the PCM, thus, allowing a minor increase in the cell volume. Interestingly, by reducing the FCD of the PCM below that of the cell, the cell experienced a hypotonic challenge with inflow of fluid and a substantial volume increase. Parabolic behavior in the pericellular fibril strain may be explained by the interaction between the osmotic pressure, fibrils, and cell. As the pericellular FCD was lower than the FCD of the cell, the fluid inflow and substantial increase in the cell volume increased the average fibril strain in the PCM. On the other hand, with high values of the pericellular FCD, the increased osmotic pressure increased the pre-stress of the collagen fibrils in the PCM. Changes in the absolute cell volume and pericellular fibril strain were amplified in the ACLT model, which incorporated weaker collagen fibrils compared to the CNTRL model.

In comparison between ACLT and contralateral groups, a 2 MPa stress followed by a force relaxation was applied. This led to different tissue strains between the groups as cartilage properties were altered. As stated above, especially the reduced superficial collagen fibril modulus increased both global and local strains, which further led to the increased cell volume. However, under a constant strain (16%), changes in the ECM composition observed in the early OA (i.e., a decrease in the collagen fibril modulus, a decrease in FCD, and an increase in the fluid fraction [1–4]) reduced normalized cell volume. Since collagen is known to control tensile stiffness of cartilage, this suggests that weakened or fibrillated ECM may expose cells to smaller tensile, transversal forces, and strains. On the other hand, the FCD of the ECM has a major role in the swelling properties and equilibrium response of cartilage [53, 54]. Therefore, the decrease in the cell volume as a result of the reduced extracellular FCD content may be explained by the reduced equilibrium stiffness of the ECM and subsequently reduced compressive and tensile forces exerted on the chondron. The parametric analyses of the effect of the PCM properties produced the same conclusions regardless of the loading protocol (constant stress followed by force relaxation or constant strain); only a decrease in the pericellular FCD caused an increase in the cell volume in mechanically loaded cartilage.

In the experiments and in the model, equilibrium states were assumed before compression and after 20 min of the relaxation. Thus, the negligible effect of the fluid fraction on the normalized cell volume during tissue compression was observed. In the future, experimental tests should be conducted to examine cell deformation behaviour under physiological, dynamic tissue loading, and computational modeling should be applied to reveal similar causes for cell deformation behavior as observed here for steady-state conditions.

In the experimental measurements, cells in the ACLT joint cartilage were compressed 7 (4 week ACLT) and 6 percentage points (9-week ACLT) less compared to the contralateral joint cartilage. However, in the FE model representing the ACLT joint cartilage, the cell was compressed 9 percentage points more in the ACLT cartilage model compared to the contralateral cartilage model (Table 3). The absolute cell strain values were also higher in the model than in the experiments. This is consistent with a recent study, suggesting that the cell stiffness *in situ* may be substantially greater than that measured *in vitro* [20], and that the PCM properties may have altered substantially more in the experiments as expected. This suggests that our choices for the material parameters of the PCM and cell may not have been fully accurate. Additional analyses showed that a 50% decrease in the pericellular FCD caused an axial compressive cell strain of 36% and a transversal cell strain of 35%. These approached slightly the values observed in the experiments (Table 3). Moreover, cell properties for instance due to their hyperactivity in OA cartilage [2, 55–58] may have increased the cell stiffness in the experiments. This may have further increased the difference in cell strains between the experiments and the model. However, differences in the absolute cell strain values do not affect the general behavior of cells and conclusions of this study.

Due to the axisymmetric model, the cell was positioned at the center of the indenter, thus, local ECM strains and cell morphology were also defined at that location. In the experiments, the analyzed cells were located arbitrarily under the indenter. This could partly explain the differences in the absolute values of cell strains between the FE models and experiments. Furthermore, a true 3D geometry with the split lines of cartilage and different cell width and depth were not accounted for in the model [18]. 3D modeling would certainly enable better assessment and modeling of cell morphology [28, 59]. Especially, changes in cell width and depth under cartilage loading, and their relationship with split lines, could be studied separately. Nevertheless, the split-line implementation and real 3D geometry would not change the conclusions of this study. 

Experimental studies investigating simultaneously cell volume and synthesis have shown that ~30 and ~50% static compression of cartilage decreases cell volume and PG synthesis approximately ~30–40% and ~40–70%, respectively [13–15]. We found only a 4% decrease in the cell volume for the contralateral joint cartilage under a 16% static compression, which may indicate only a small alteration in the cell synthesis. On the other hand, the cell volume increased by 7% in the ACL transected joint cartilage. It has been earlier approximated that a change in the extracellular osmotic environment of chondrocytes from ~350 mOsm to ~280 mOsm can lead to a ~10–17% increase in the cell volume and a ~30% decrease in the synthesis rates of collagen and PGs [12, 60]. As we investigated cell volume behavior under static mechanical loading of cartilage, the cell volume increase and its relationship to cell synthesis may not be directly related to the results obtained from osmotic loading experiments. In fact, likely the experimentally detected cell volume increase in the present study is a result of a failure in the protection mechanism of the PCM, exposing cells to abnormal signals and even death.

The study by Huyghe and Wilson [61] indicated that the current formulation of the chemical expansion stress (see (7)) does not fulfill the 2nd law of thermodynamics. Yet, it is discussed in that paper that osmotic pressure alone is not able to describe the swelling behavior of cartilage. Consistently, our FE simulations suggest that neglecting the chemical expansion stress reduces the increase in cell volume in OA cartilage (ACLT model) and, thus, increases the difference between the model and experimental results. However, it does not change the conclusions of this study. It would be important to find an exact formulation for this property in order to model the cell and cartilage swelling behavior accurately.

Several studies have suggested that the PCM may protect chondrocytes from external stresses [8–10, 18, 19, 24]. Changes in the ECM and PCM properties, due to early OA, may weaken this protective ability, and this may expose chondrocytes to abnormal external stresses and strains. The results of the present study suggest that there are various simultaneous processes in cartilage during OA progression, especially PG loss and alterations in the collagen network [1–4], which contribute to the regulation of the cell volume and deformation behaviour. Increased tissue deformation as a result of collagen fibrillation, and subsequently reduced collagen fibril stiffness, and reduced pericellular FCD are suggested to be the main modulators of the cell volume, presumably affecting cell biosynthesis and viability [11–15]. This suggests that the FCD may have an important role in cell volume regulation, and the contribution of the FCD to the pre-stress of the collagen network may be important in preventing “overswelling” of cells. If this state of changed cell volume persists, it may cause progressive changes or even degradation in the PCM and ECM, leading to the progression of OA. Therefore, it appears that if collagen could be regenerated quicker, or cells could accelerate the production of FCD in early OA (which has been suggested to occur in OA [2, 55–58]), early changes in OA could possibly be reversed. 

## 5. Conclusions

The results of our study suggest that the loss of the FCD in the PCM and fibrillation of the superficial ECM cause cell volume increase in mechanically compressed early OA cartilage. Increased cell volumes may alter chondrocyte biosynthesis, or even cause cell death, thus exposing the cartilage to matrix degradation and the progression of OA.

## Figures and Tables

**Table 1:** Mechanical, structural, and compositional parameters of the ECM, PCM, and cell of the reference model. Parameters were implemented into the fibril-reinforced poroelastic swelling model of articular cartilage.

	ECM	PCM	Cell
Mechanical parameters			
*E*_*f*_ (MPa)	10	0.1 ∗ *E*_*f*_^ECM^	—
*E*_*m*_ (MPa)	0.505	0.0505	0.017
*υ*_*m*_	0.15	0.15	0.30
*k* (10^−15^ m^4^/Ns)	1.9	1.9	1900
Composition			
*n*_*f*_	0.85 − 0.15*z*	0.85	0.61
*c*_*F*_ (mEq/mL)	0.11 + 0.09*z*	0.14 + 0.12*z*	0.08
Zone depth			
Superficial	0.07	—	—
Middle	0.18	—	—
Deep	0.75	—	—

**Table 2:** Parameters implemented in the parametric fibril reinforced poroelastic swelling models. Parametric analyses were conducted by varying one parameter, while the other parameters were kept constant. CNTRL: contralateral, ACLT: anterior cruciate ligament transected.

Model	Collagen stiffness (*E*_*f*_) (MPa)			Fixed charge density (*c*_*F*_) (mEq/mL)			Fluid fraction (*n*_*f*_)		
	ECM	PCM	Cell	ECM	PCM	Cell	ECM	PCM	Cell
Experimental loading protocol^†^:									
CNTRL model	Table 1	Table 1	Table 1	Table 1	Table 1	Table 1	Table 1	Table 1	Table 1
ACLT model	7.5	0.1 × *E*_*f*_^ECM^	Table 1	0.08 + 0.12*z*	0.11 + 0.09*z*	Table 1	0.9 − 0.2*z*	0.90	Table 1
Parametric CNTRL PCM model	Table 1	[0.01,1] × *E*_*f*_^ECM^	Table 1	Table 1	[0.02,0.18]	Table 1	Table 1	[0.50,0.95]	Table 1
Parametric ACLT PCM model	7.5	[0.01,1] × *E*_*f*_^ECM^	Table 1	0.08 + 0.12*z*	[0.02,0.18]	Table 1	Table 1	[0.50,0.95]	Table 1
Parametric ECM model	[7.5,10]	0.1 × *E*_*f*_^ECM^	Table 1	**	Table 1	Table 1	[0.50,0.90]	Table 1	Table 1
Constant strain:									
Parametric ECM model	[2.5,10]	0.1 × *E*_*f*_^ECM^	Table 1	**	Table 1	Table 1	[0.50,0.90]	Table 1	Table 1
Parametric PCM model 1	Table 1	[0.01,1] × *E*_*f*_^ECM^	Table 1	Table 1	[0.02,0.18]	Table 1	Table 1	[0.50,0.95]	Table 1
Parametric PCM model 2	7.5	[0.01,1] × *E*_*f*_^ECM^	Table 1	Table 1	[0.02,0.18]	Table 1	Table 1	[0.50,0.95]	Table 1
Parametric PCM model 3	5.0	[0.01,1] × *E*_*f*_^ECM^	Table 1	Table 1	[0.02,0.18]	Table 1	Table 1	[0.50,0.95]	Table 1
Parametric PCM model 4	2.5	[0.01,1] × *E*_*f*_^ECM^	Table 1	Table 1	[0.02,0.18]	Table 1	Table 1	[0.50,0.95]	Table 1

^†^In the experimental loading protocol the strain was kept constant after a loading stress of 2 MPa was reached.

**In the parametric ECM model 5 different depth-dependent FCD distributions were implemented (*c*_*F*_ = 0.011 + 0.009*z*, *c*_*F*_ = 0.06 + 0.045*z*, *c*_*F*_ = 0.11 + 0.09*z*, *c*_*F*_ = 0.165 + 0.135*z*, and *c*_*F*_ = 0.22 + 0.18*z*). *z* is normalized cartilage depth (0 = surface, 1 = bottom).

**Table 3:** Local ECM and cell strains, and cell volume in contralateral (CNTRL) and anterior cruciate ligament transected (ACLT) rabbit knee joint cartilages 4 weeks (Turunen et al. [22], *N* = 8 pairs of joints, *n* = 77 in CNTRL group cells, *n* = 79 in ACLT group cells) and 9 weeks (Han et al. [23], *N* = 4 pairs of joints, *n* = 48 cells in both groups) after ACL transection. Experimental data is presented as mean ± standard error of mean.

	Turunen et al. [22]		Han et al. [23]		Simulations	
	CNTRL	ACLT	CNTRL	ACLT	CNTRL	ACLT
Local axial ECM strain (%)	43 ± 4	47 ± 4	27 ± 5	38 ± 4**	30	39
Local transverse ECM strain (%)	17 ± 4	20 ± 3	8 ± 3	14 ± 3*	6	11
Change in cell height (%)	24 ± 2	17 ± 2**	18 ± 2	12 ± 2	29	38
Change in cell width^†^ (%)	13 ± 1	22 ± 1***	7 ± 1	12 ± 1**	20	36
Change in cell volume (%)	−5 ± 1	24 ± 4***	−8 ± 2	8 ± 2**	−4	7

^†^In the experiments, cell width refers to an average width and depth.

*P < 0.05, **P < 0.005, ***P < 0.001 between CNTRL and ACLT groups.
